# Supplementary material for: Effect of intramedullary nail stiffness on load-sharing in tibiotalocalcaneal arthrodesis: A patient-specific finite element study
Source: PLoS One. 2023 Nov 16;18(11):e0288049. doi: 10.1371/journal.pone.0288049 (PMC10653524; doi:10.1371/journal.pone.0288049)
Supplement: S1 File — This flow chart is used by the Colorado Multiple Institutional Review Board to determine whether a study falls under the classification of human subjects research. Because cadaveric specimens are not alive, the use of a donated specimen in our study did not qualify as human subjects research. (PDF) [file pone.0288049.s001.pdf]

Decision Tree for Determination of Human Subjects Research

Is your project research?

The intent of the proposed project:

- Innovative **medical therapy** to improve the health of an individual patient
- **Operational activity** of an organization (e.g. customer service initiative, teaching evaluation, disease outbreak investigations, auditing)
- **Journalism**
- **Political poll**
- **Oral history**, **biographies**, or **medical case history** that is not generalizable beyond that individual
- **Classroom or training activities** where the only objective is to teach a student proficiency on a topic
- **Quality Improvement or Quality Assurance**
  - o program initiated by an institution to compare performance to established standards or measure the value of a program to its participants

    AND
  - o information will only be distributed to those directly involved in its planning, management and implementation

If yes, proposed project is not research

→ **NOT HUMAN SUBJECTS RESEARCH- COMIRB Submission Not Required**

The intent of the proposed project:

- **Systematic investigation** intended to develop or contribute to **generalizable knowledge**.
  - Systematic investigation means carried out using step-by-step procedures organized according to a set of interrelated ideas or principles.
  - Generalizable knowledge may include**:**
    - Benefits that extend beyond immediate population- to society, other researchers, scholars, or practitioners in field
    - Drawing conclusions, testing or generating a hypothesis
    - Publication or presentation to inform the field of study
    - Contributes to a theoretical framework or body of knowledge
- **Test a new device, product, drug or biologic material**

If YES, proposed project is research

Are human subjects involved?

The proposed project involves:

- **Individuals that are not living**
  - o cadavers
  - o autopsy specimens
  - o data or information on deceased individuals*
- **Animals, animal specimens or animal cell lines****

* PHI requires clearance from Privacy Officer

** IACUC review required

If yes, human subjects not involved → **NOT HUMAN SUBJECTS RESEARCH- COMIRB Submission Not Required**

The proposed project involves:

- Data about or specimens from one or more living individuals

YES, human subjects involved

→ **Submit to COMIRB for determination**
