# Supplementary material for: Effect of intramedullary nail stiffness on load-sharing in tibiotalocalcaneal arthrodesis: A patient-specific finite element study
Source: PLoS One. 2023 Nov 16;18(11):e0288049. doi: 10.1371/journal.pone.0288049 (PMC10653524; doi:10.1371/journal.pone.0288049)
Supplement: S2 File — This standard operating procedure from our laboratory provides detailed instructions on how to run finite element analysis with an intramedullary nail digitally implanted in a patient-specific ankle model. Instructions on how to activate the nickel titanium compressive element in a pseudoelastic IM nail are included. (PDF) [file pone.0288049.s002.pdf]

<u>Finite Element Analysis of Pseudoelastic Compression Generating Intramedullary Nail for Ankle Arthrodesis</u>

Standard Operating Procedure by Ryan Anderson

The procedure presented illustrates how to setup and run the finite element analysis of the DynaNail and bone ankle fusion model. It is assumed that the input file has been successfully exported from ScanIP with the mesh and material property assignment. If an individual is interested in applying the same method to a similar model containing a nickel titanium (NiTi) material, it is useful to have a material placeholder for the NiTi region. Furthermore, if using a manual locking material method, like the one presented here, it is important to have a region of locking material within the ScanIP model that can be meshed and written to the input file. This makes things significantly easier to maintain the input file format and element-numbering format. It is assumed that the reader of this procedure has common knowledge of finite element analysis and ABAQUS software. ABAQUS documentation can be consulted for tutorials and more information if necessary.

<u>How to manually add locking elements:</u>

Directly following the output of the input file from ScanIP we will manually modify the input file to include locking elements. We first start by opening the input file in ABAQUS.

**Any rotation or movement of the model within ABAQUS can be obtained by simultaneously holding down CTRL+ALT. Left clicking and dragging will cause rotation while holding down the scroll wheel and dragging will cause translation.

Open ABAQUS CAE

File ➔ *Set Work Directory…* ➔ choose folder directory

**It is important to keep all model files together in one folder for organization and to ensure files are not lost. If running various CAE files, keep them separated into individual folders.

File ➔ *Import* ➔ *Model* ➔ choose ScanIP export file

Make sure to change *File Filter* from *Abaqus/CAE Database (.cae)* to *Abaqus Input File (*.inp, *.pes).* This will make any .inp files show up in the finder window.

Using *display groups,* create a set that can display the outer jacket of the nail as well as the NiTi rod. These should now be the only groups visible. By using a view cut and zooming into the proximal region of connection for the NiTi rod (locking element location), a set of nodes can be selected and used for the manual locking elements. On the proximal head of the NiTi rod, choose 3 nodes centrally located on the surface that connect to make a base (Figure 1). Use the query tool (Tools➔Query➔Point/Node) to find the node numbers and write them down. Next, look at the nail jacket side and find 3 nodes that make a base as close to axial as possible to the first base. Query the nail jacket nodes and write them down. On each base, pick one node that will act as the point for the element that faces each other. Now you should have a list with 4 nodes (3 base and 1 point) for element 1 and another 4 nodes (3 base and 1 point) for element 2. If correct,

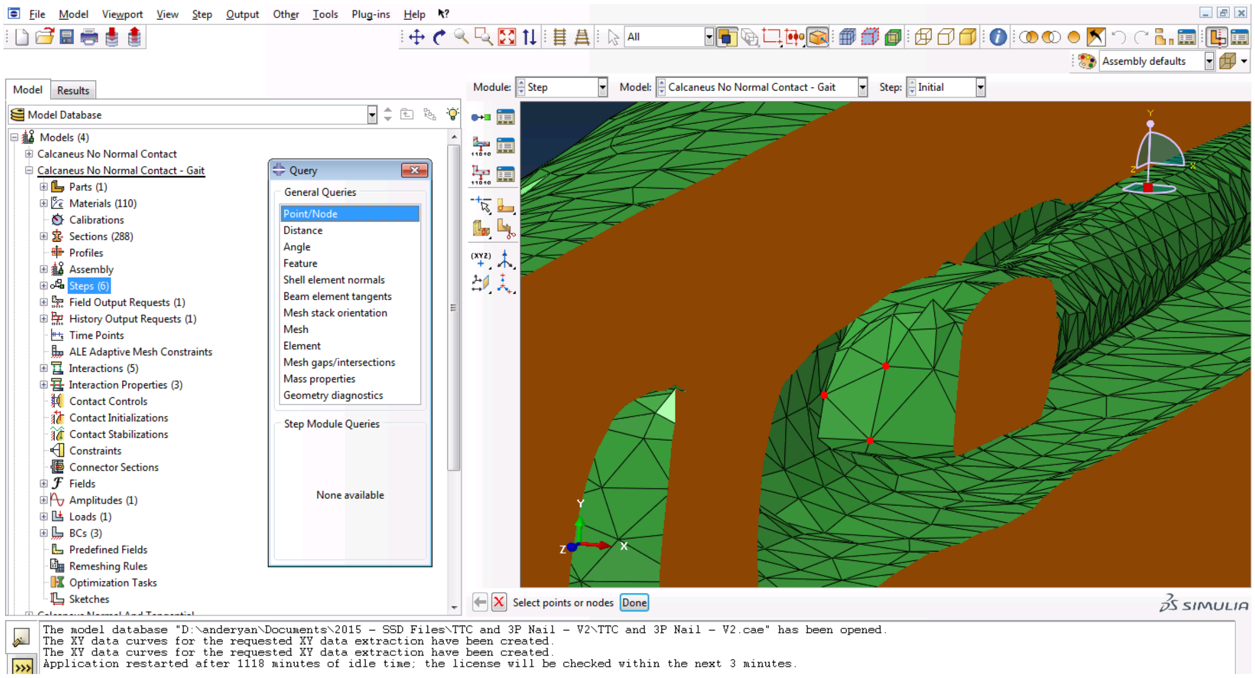

Figure 1: NiTi Base

each element will have 4 different numbers but the point should correspond to a number in the other element's base.

We can now close out of ABAQUS . Make a copy of the original input file that we can use for modification. Using a text editor, open the copied input file (.inp) and use the search function to find the element list for the locking material. Since a mask was created in ScanIP named "Locking Material" and consisted of volume, a search for "Locking Material" in the input file brings up a few results. Refer to Figure 2 to ensure proper position within the text. A list of element numbers (first number) in numerical order and their corresponding nodes (last 4 numbers) should exist for the "Locking Material". Delete the entire list of elements inside the

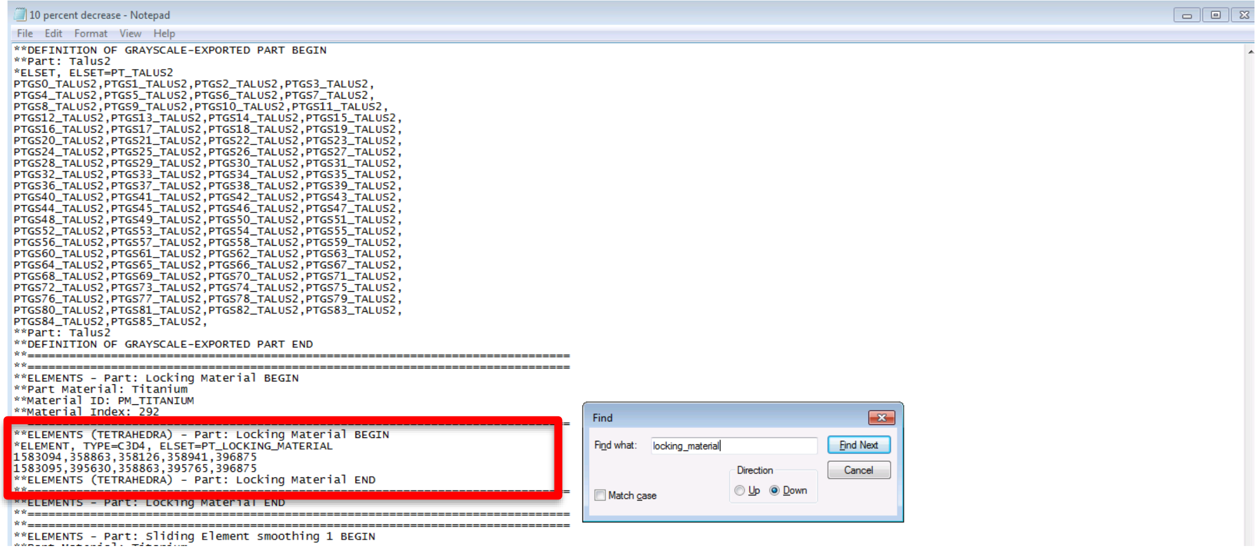

Figure 2: Modifying Input File

“Locking Material” list EXCEPT the first two elements. Now, replace the last 4 numbers in each element with the nodes written down from the model query. See Figure 2 for final product. Order does not matter at this point.

**It should be noted that the elements used in this model are tetrahedral in shape. If a different shape is chosen, the number of nodes necessary for an element will change. Additionally, due to the geometry of tetrahedron elements, large distortion can occur easily. This can arise from extreme rotation or extreme translation. Depending on the size of the element base and the length of stretch (in this case 6 mm stretch), distortion can arise. Therefore, as element size is decreased (higher mesh density), the smaller the locking elements base will be and thus higher likelihood of distortion. Distortion can lead to convergence problems so keep this in mind when choosing element size and type.

Save the modified input file (.inp) and open ABAQUS again. Follow the same steps above to input the model except this time, navigate to the modified input file.

THIS IS IMPORTANT: I have not been able to find a definitive pattern to list the node numbers correctly; thus, ***you have to*** check the locking elements once the modified model is imported. If the node numbering is out of order, the element will look like it doesn’t have a face (empty) and this means it has no volume. A figure of a good one is in Figure 3L and a bad element in Figure 3R. **If you forget this step, all the work to follow will be for nothing because the analysis pre-processor will abort due to negative or low volume element detection.** This means that you have to go back and modify the input file again and start the ABAQUS model from scratch. So, if you check the locking elements and they do not have volume, exit out of ABAQUS and go back to the modified input file. For each locking element, take the second node and swap it with the first node (see Figure 4). This usually does the trick but sometimes you have to try a different position combination. Now go back to ABAQUS and see if the element looks correct. Once both elements have volume, you can proceed.

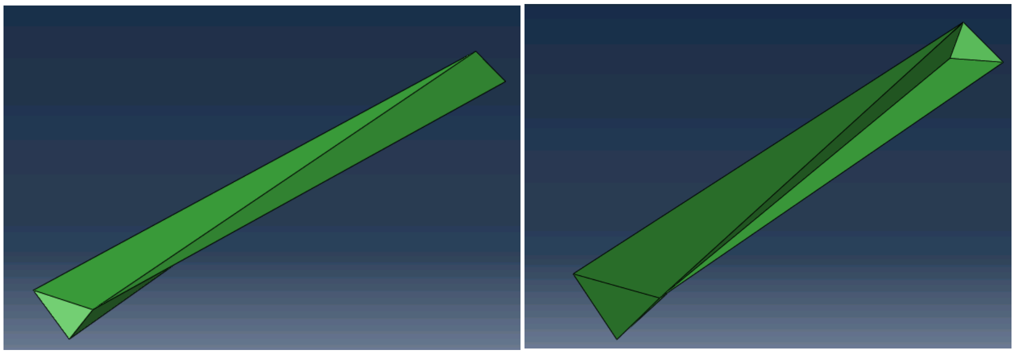

Figure 3: Good Elements (Left) & Bad Elements (Right)

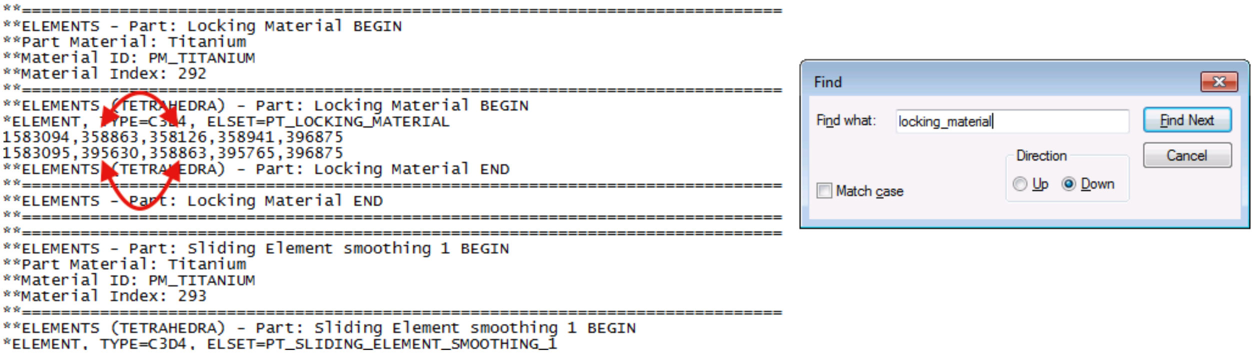

Figure 4: Swapping Node Numbering

Before starting to add parameters to the model, save. Make sure the extension is correct (.cae). When saved, it will hold all of the steps and jobs created for this model.

At this point, delete Model-1 within the model-tree since this is the default model created when opening ABAQUS. We can now expand on the model we imported within the model-tree.

From the model tree ➔ expand *Materials* ➔ Find NiTi material placeholder ➔ RIGHT CLICK ➔ *Edit*

Within the *Edit Material* window,

General ➔ *Depvar*

In the *Depvar Material Behavior* section, the *Number of solution-dependent state variables* should be changed from 0 ➔ 24. *Variable number controlling element deletion* can remain as 0.

Within the *Edit Material* window,

General ➔ *User Material*

User material type: **Mechanical**
Mechanical Constants: 40270, 0.33, 35218, 0.33, 0.04, 11.53, 385, 400, 0, 8, 207, 200, 0, 0.04, 0

**After entering the first mechanical constant, ENTER will create a new row. Likewise, right clicking the row number can insert and delete rows. Make sure to only have 15 material constants (delete row 16 if necessary) like in Figure 5. Mechanical constants listed above were specific to the DynaNail ankle fusion model and can be modified for a different material response. Refer to ABAQUS superelastic built in UMAT literature for more information.

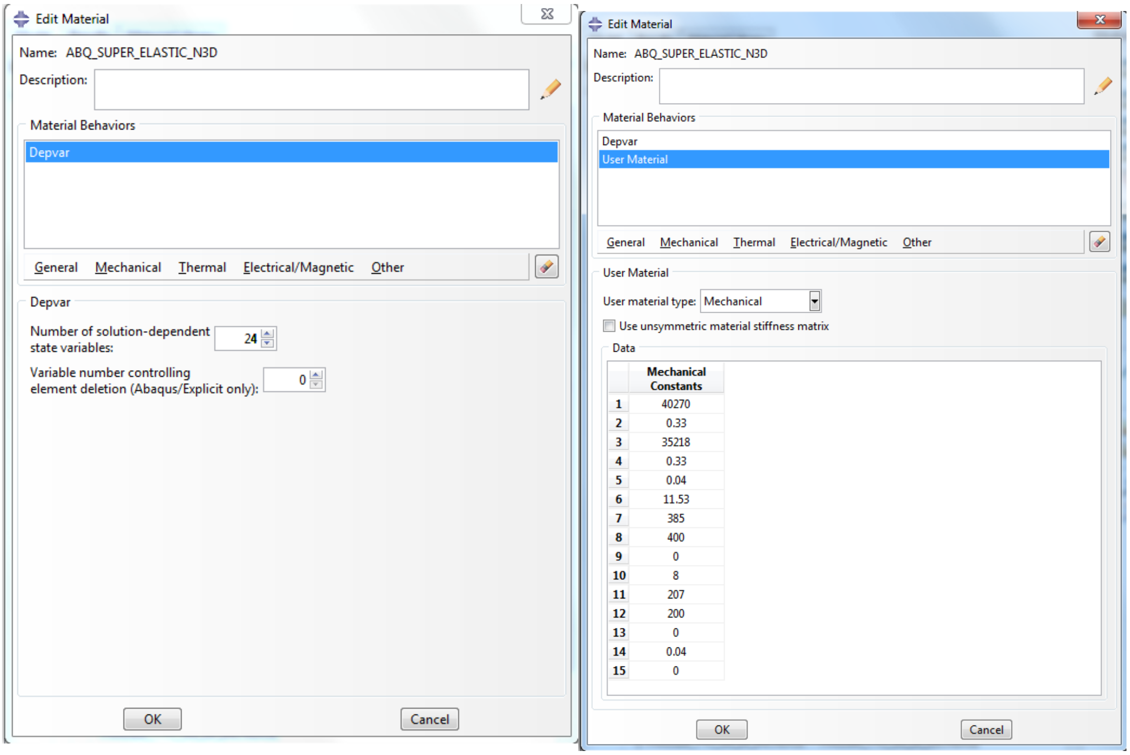

Figure 5: NiTi Material Settings

Rename the material by right clicking on the material name in the material-tree to:

ABQ_SUPER_ELASTIC_N3D

**This is explained in ABAQUS literature. The name needs to be exact, it is used to trigger the built in UMAT. The last 3 letters can be changed to N2
D if working on a 2D model or N1D if working on a 1D model.

Under the model-tree, expand *Sections*. Locate the section that pertains to the NiTi material. In this case, it is named SECTION-291-PT_COMPRESSIVE_ELEMENT_FROM_CAD.

RIGHT CLICK ➔ *Edit*

An alert will render that says the material that was previously assigned does not exist. Hit *Dismiss*.

Under *Edit Section* window➔ *Material:* ➔ Choose ABQ_SUPER_ELASTIC_N3D from the listed materials ➔ *OK* (See Figure 6)

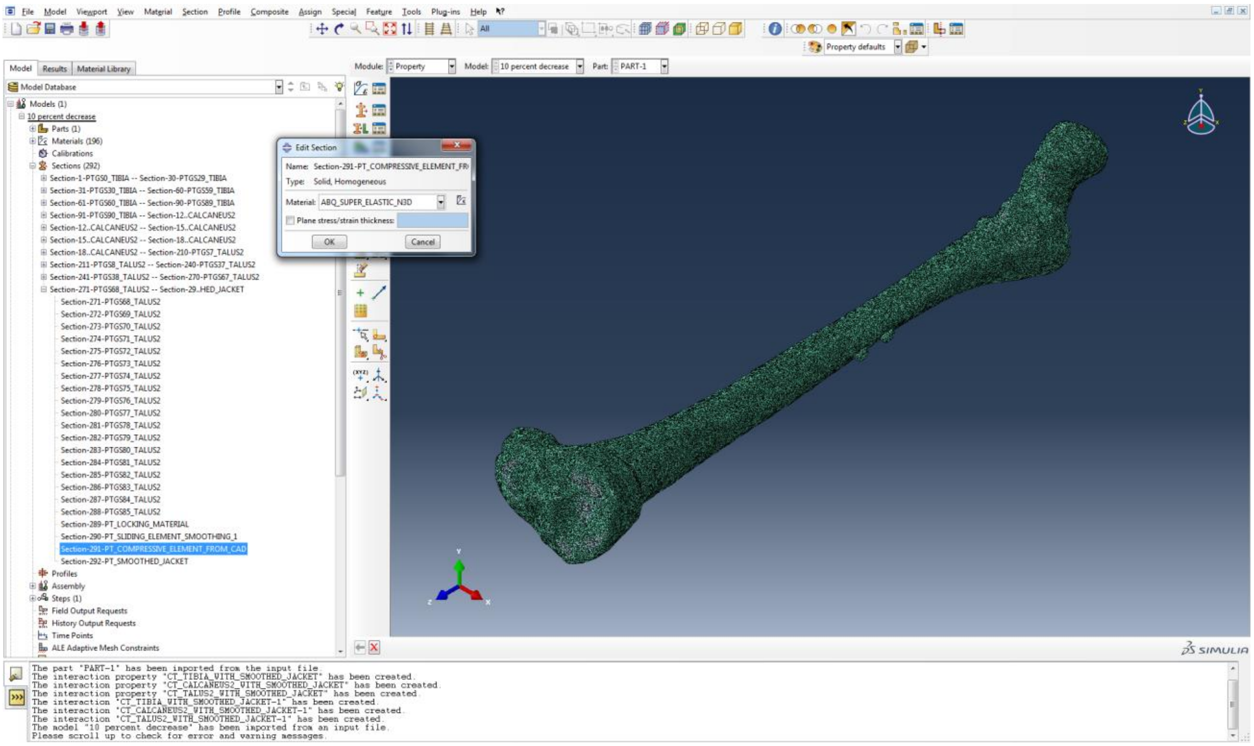

Figure 6: Reassigning Material Properties to Section

<u>STEP 1 - Stretch</u>

Now we need to change to the *Step* Module. Since we will be creating a step, double click on *Steps* in the model-tree and a *Create Step* window will appear.

Name this step “Stretch”. Procedure type is General – *Static, General*. Hit *Continue…*

In the *Edit Step* window (See Figure 7):

➔ *Basic* ➔ *Time Period* = 1
➔ *Nlgeom* = *On*
➔ *Automatic Stabilization* = *Specify dissipated energy fraction*
* Use default settings for *dissipated energy fraction*
➔ *Include adiabatic heating effects* = Unchecked

➔ Incrementation ➔ *Type* = *Fixed*
➔ *Maximum number of increments* = 10,000
➔ *Increment size - Minimum* = 1E-10
\- Initial = Maximum = 1

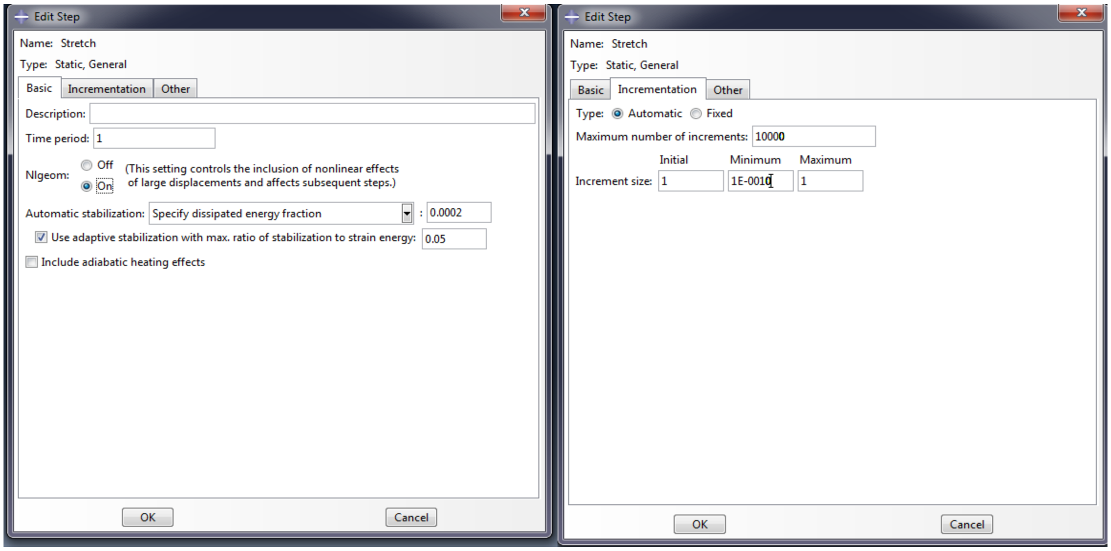

Figure 7: Step Preferences

**Nlgeom takes into account non-linear geometry and should be used in cases where large displacement (greater than 1% strain) occurs. Since the NiTi is stretched to 6% strain, this needs to be accounted for. Automatic stabilization adds a certain fraction of energy to the system to allow a solution. Total added energy can be monitored for the results to ensure the amount is not large in comparison to total energy of the system. This model successfully converged using an initial and maximum increment set to 1, however, it has been documented that a smaller maximum increment size (~0.25) can help with convergence at the junction of the start or end of the transformation zone (M. Azaouzi et al. 2012).

Now that we have 1 step created, we have to modify the solution controls to allow convergence of the solution (Figure 8).

At the top of the window: *Other* ➔ *General Solution Controls* ➔ *Manager…*

Select “Stretch” ➔ *Edit…* ➔ *Continue…*

Change *Propagate from previous step* to *Specify*.

Time Incrementation tab ➔ First *More* from the top ➔ I_A ➔ Change 5 to 100 ➔ *OK*

**This little trick allows more than the default of 5 attempts when the solver is making cut backs on the incrementation. During development of the model, it was observed that a solution was unable to converge; but with this trick, the model just needed one more cut back attempt during the relaxation of the NiTi for a total of 6 cutbacks. Now that the solution controls are modified for the first step, the settings will propagate for the rest of the steps.

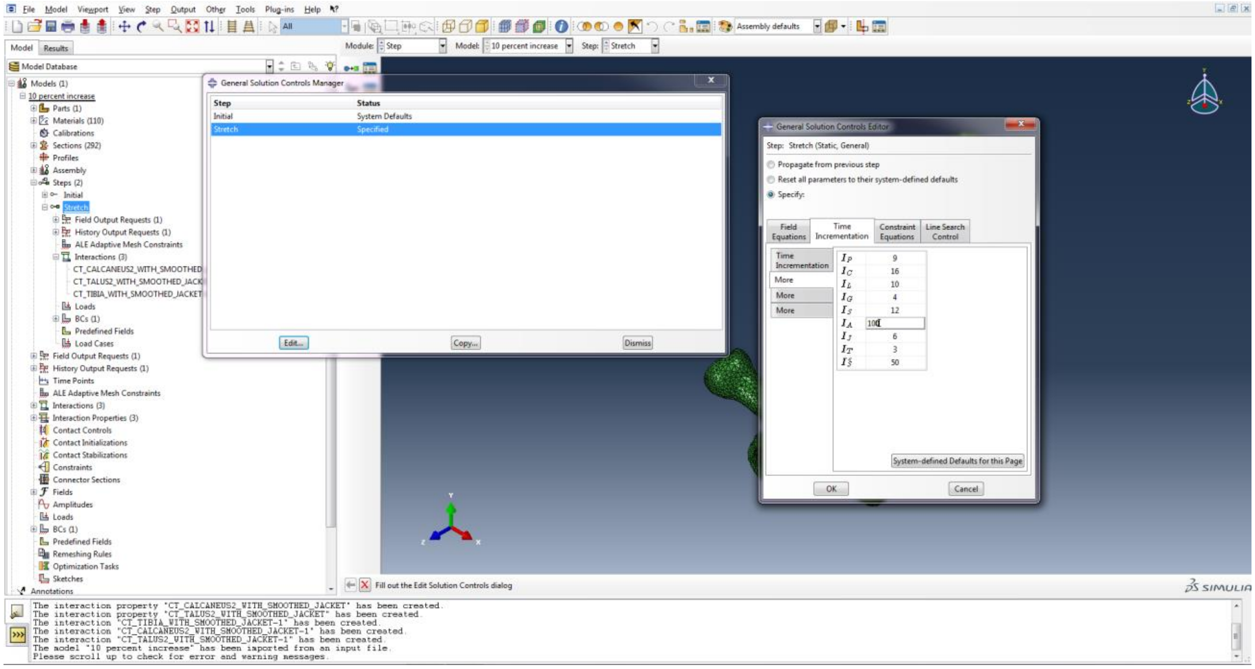

Figure 8: General Solution Controls

<u>*Boundary Conditions*</u>

Now let's get to applying boundary conditions. First, lets expand upon the *Initial* step within the Step-tree. Double clicking on the *BCs* we are now prompted to assign a boundary condition.

Name this boundary condition "Pin Sliding Element" and this will be a *Mechanical - Symmetry/Antisymmetry/Encastre* type. Now hit *Continue …*

Now, select the element region of the whole sliding element. The easiest way to do this is to use *display group manager*. By selecting the sliding element set, the selection can be saved as a display group and then solely plotted in the viewport. Select the elements and hit *Done*.

In the type of constraint, select *PINNED (U1 = U2 = U3 = 0)*, Hit *OK*.

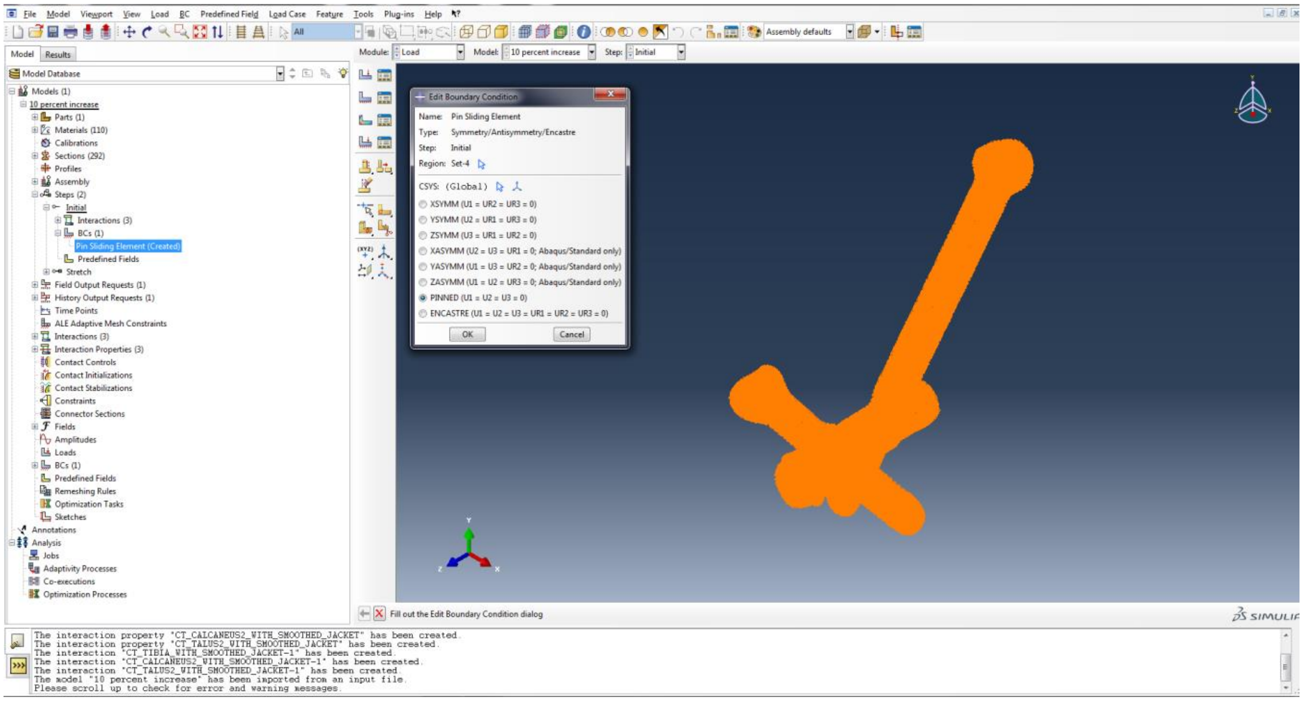

Figure 9: Pin Sliding Element

Back to the "Stretch" step, create a boundary condition that displaces the NiTi. Double click *BCs*, name this "Displace NiTi" and this will be a *Mechanical – Displacement/Rotation* type. Hit *Continue …*

Using *display group manager*, display just the compressive element. One end should appear to be pinned already (nodes are shared between sliding element and compressive element at attachment). Make the element selection at the other end of the compressive element for displacement. Hit *Done*. Now we will check the boxes for U1, U2, and U3. U3 is our axial displacement so enter 8 here while the others will remain zero. Hit *OK*.

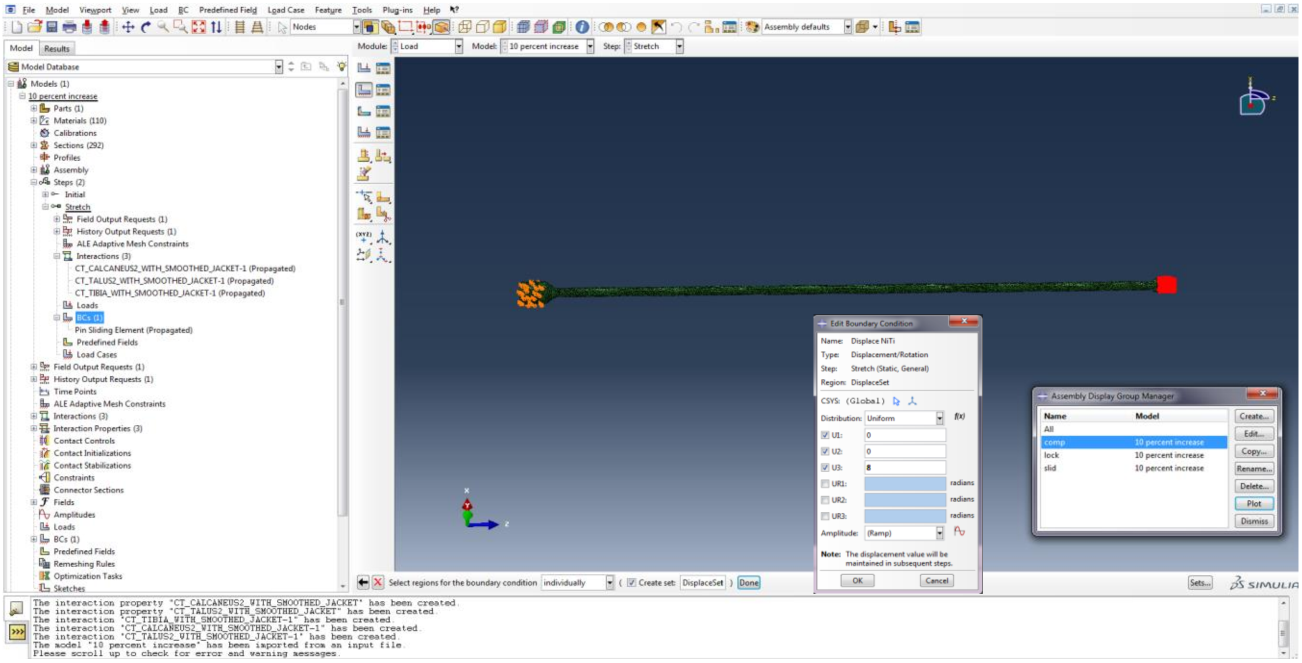

**We do not need to check UR1, UR2, nor UR3 since this model does not have rotational degrees of freedom.

Interactions

In an attempt to decrease computational taxation, we will remove locking elements and bone elements in the NiTi "programming" steps. To remove the locking elements and bone, we need to create 2 new interactions. Within the "Stretch" step-tree, double click on *Interactions*. We will name the first interaction "Bone Model Change" which we select *Model change* and hit *Continue…*

Within the *Edit Interaction* window (Figure 10) we select:

Definition: *Region*
Region Type: *Elements*
Region: click the arrow and it will allow a selection to be made (select all bone elements – as can be seen I've named the element set "BoneModelChange")
Activation state of region elements: *Deactivated in this step*
Hit *OK*

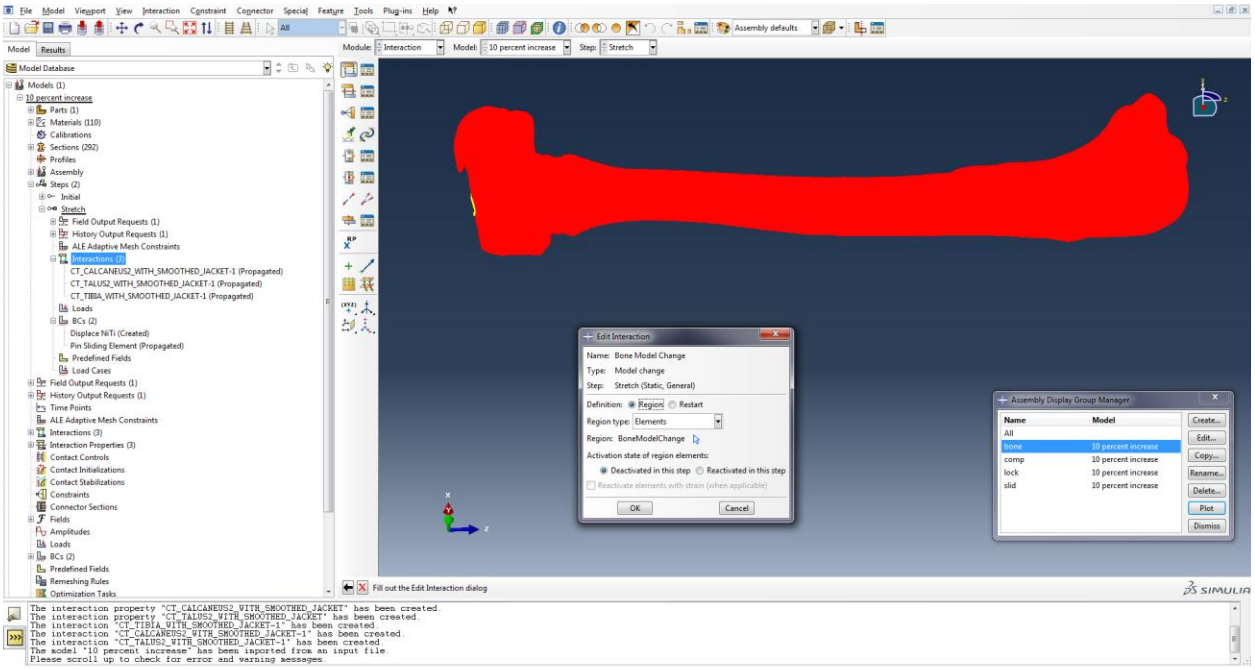

Figure 10: Bone Model Change

We will repeat this process for the locking elements. Double click *Interactions* and name this one "Locking Material Model Change" which we select *Model Change* and hit *Continue …*

Definition: *Region*
Region Type: *Elements*
Region: click the arrow and it will allow a selection to be made (select all locking elements)
Activation state of region elements: *Deactivated in this step*

Hit *OK*

**There is a possibility that a display group could be created that encompasses all bone elements *and* the locking elements that can be deactivated together for 1 single model change interaction. However, this was not tested.

If you have not already, expand the *Interactions* tree within the "Stretch" step. There should now be two model change interactions as well as any contact interactions defined within ScanIP. Any contact properties that pertain to elements deactivated (bone or locking material) need to be also deactivated or the solution will diverge. In this case, 3 contact properties exist and each can be deactivated as follows:

Right click on the contact interaction ➔ *Edit*

In the *Edit Interaction* window, deselect the *Active in this step* box at the bottom of the window (Figure 11).

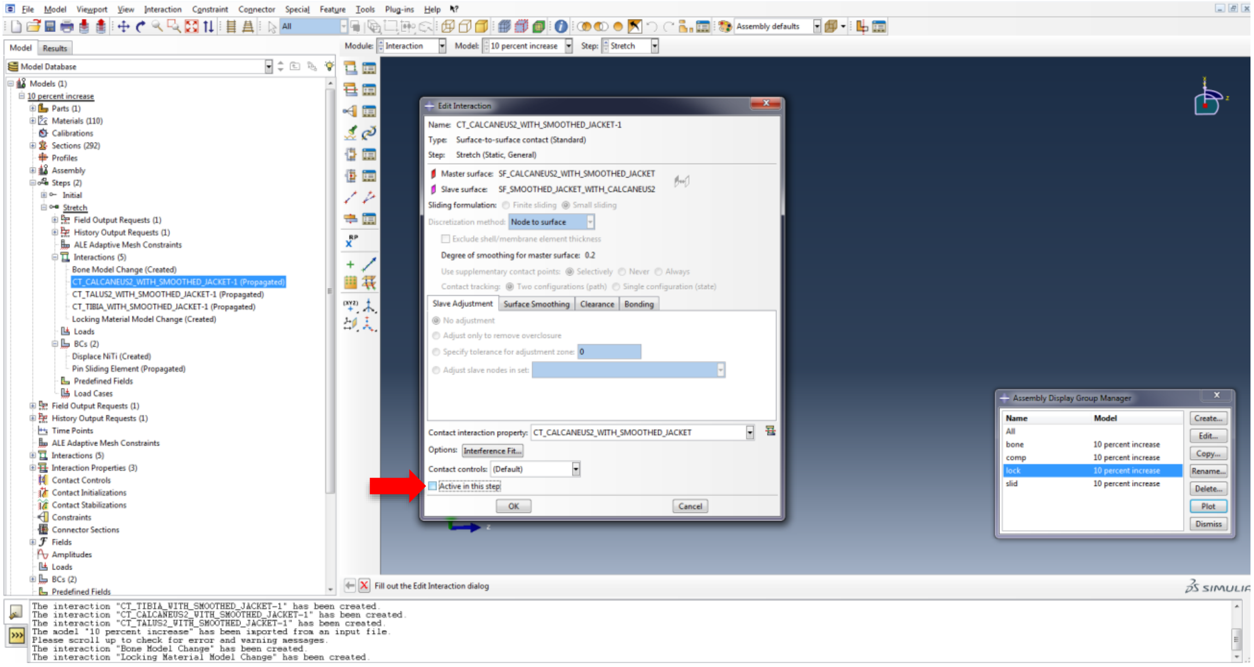

Figure 11: Deactivating Contact Interaction

We also need to ensure contact properties are correct. In the model-tree, expand upon the *Interaction Properties*. For this model, contact properties were defined between the nail jacket and all 3 bones. Therefore, the settings should be replicated for all three bones. The default tangential property needs to be changed to *Frictionless* and then a normal contact should be added. The normal contact should be *Hard* contact.

STEP 2 - Relax

Moving onto Step 2, double click the *Steps* section in the model-tree and we create a new step called “Relax”. In the *Edit Step* window, use the same settings prescribed for the “Stretch” step. The only modification necessary in this step is to modify the “Displace NiTi” boundary condition.

Boundary Conditions
Double click the “Displace NiTi” BC ➔ In the *U3:* box we replace 8 with a 6. ➔ Hit *OK*

STEP 3 - Reactivate

Step 3 is created just like previous steps with identical settings.

Interactions
We first start this step by modifying the interactions. Expanding the *Interactions* section, we can right click and edit the model changes (bone and locking material). The only option available for modifying is to switch from *Deactivated in this step* to *Reactivated in this step*, which we will do for both. The box that becomes available to *reactivate elements with strain* will be left unchecked.

**We want to reactivate the locking elements without strain so that they are now in a relaxed state but locking the NiTi rod in the “programmed” state.

Now that the bone will be placed back in the model, contact interactions should now be activated. Right click, edit, and now check the *Active in this step* box for all contact interactions.

Boundary Conditions
The displacement boundary condition on the NiTi will remain in this step but a new boundary condition will be implemented to pin the proximal tibia. Double click on *BCs* and create a *Mechanical* - *Symmetry/Antisymmetry/Encastre* type. Now hit *Continue …*

Make a selection of the proximal tibia (Figure 12) and hit *Done*. In the type of constraint, select *PINNED (U1 = U2 = U3 = 0)*, Hit *OK*.

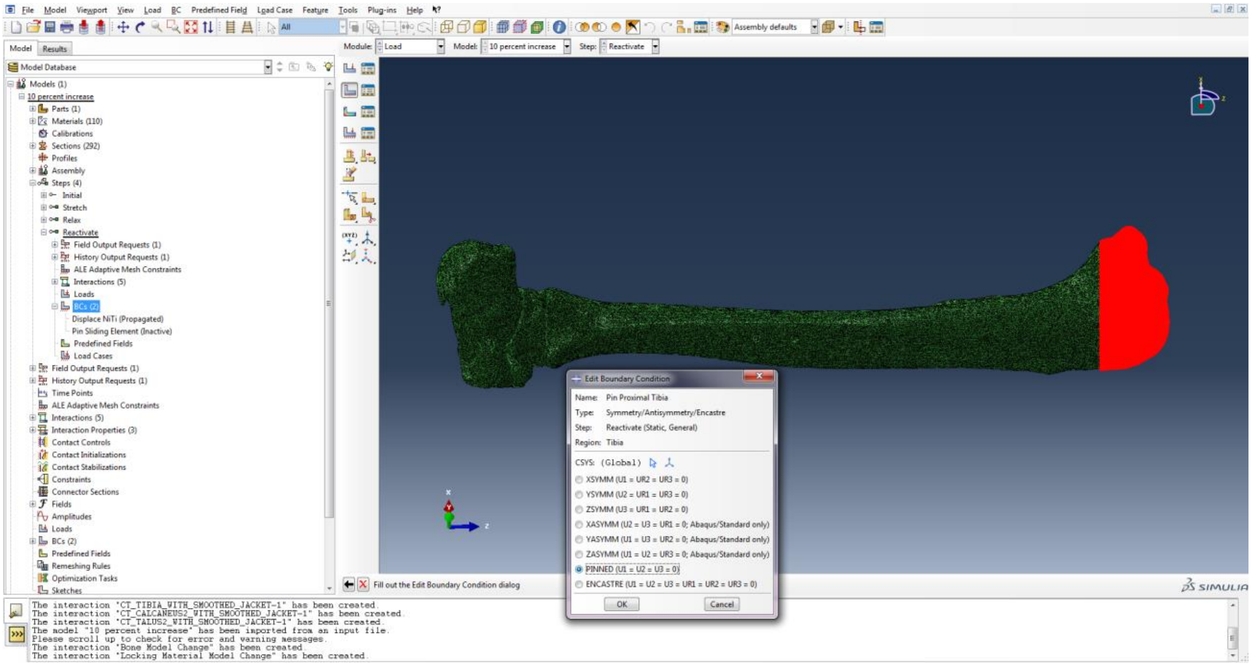

Figure 12: Pin Condition on Proximal Tibia

Now deactivate the pin condition on the sliding element by right clicking on "Pin Sliding Element" boundary condition and select *Deactivate*.

STEP 4 - Release

Let's create a "Release" step, same settings as the previous steps.

Boundary Conditions

All that is needed in this step is to deactivate the "Displace NiTi" boundary condition. Right click on "Displace NiTi" boundary condition and select *Deactivate*.

STEP 5 – Gait Load

It is time to apply a gait load to the model. We first start by defining a gait amplitude. In the model-tree, double click on the *Amplitudes* section. Name this "Gait Amplitude" and choose tabular.

Time span: *Step time*
Smoothing: *Use solver default*
Amplitude data: This is where the gait amplitude is entered as normalized (maximum is set as 1) for each entry. See Figure 13 for reference. A total of 44 rows are used.

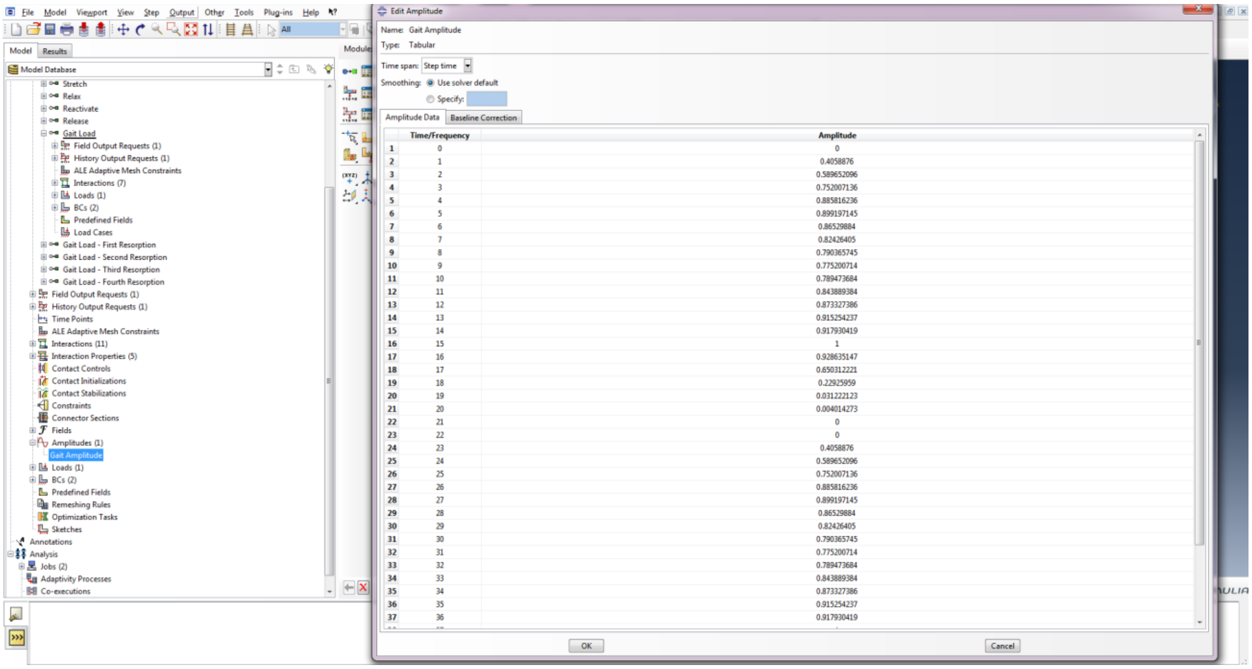

Figure 13: Gait Load Amplitude

Create a new step following the "Release" step called "Gait Load". Use all the same step parameters as the previous steps EXCEPT make the time period 44.

Expand the step-tree and double click the *Loads* section to create a new load. We are calling this "Gait" and this is a *Mechanical – Concentrated Force*. We now select the points for loading application. For this particular model, 4 nodes were selected on the distal surface of the calcaneus surrounding the nail jacket. This created an axial load as much circumferential to the nail as possible. Hit *Done*.

Distribution: Uniform
CF1: 0
CF2: 0
CF3: 280.25
Amplitude: *Gait Amplitude*
Hit *OK*.

** Remember that each node will have the same load applied. More specifically, the magnitude of CF3 is 280.25 because the maximum magnitude of the gait load (1121) was divided by 4 since we are using 4 nodes to apply the load. **Don't forget** to apply "Gait Amplitude" under the amplitude setting, or else it will default to ramp.

Submit the Job

Under the analysis-tree (below model-tree), double click on *Jobs* to create a new job. Name the job and make sure the source is the model. We will use the default job settings **except** in the

Parallelization tab make sure to check *multiple processors* (8 if using Bigfoot) and check the box for *GPGPU acceleration* (if using Bigfoot). Hit *OK*.

<u>**Analyzing the Results**</u>

This section by no means is a collective explanation for how to analyze this model or any other ABAQUS model for that matter. A few data analysis methods are presented that are not as intuitive as it might be thought.

Once the job states it is completed, right click on the job and select results. Sometimes the interface does not automatically load the output database (ODB) correctly so make sure that the ODB is correct (see Figure 14).

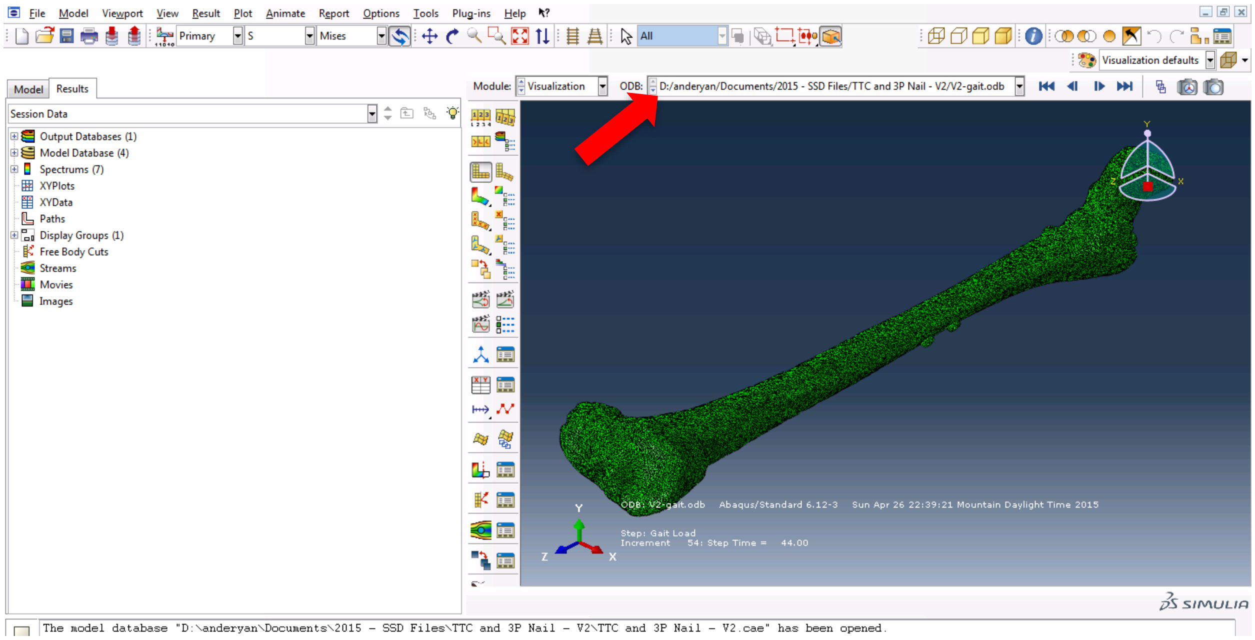

Figure 14: Opening ODB

<u>Creating a Stress-Strain plot</u>

Tools ➔ *XY Data* ➔ *Create …* ➔ *ODB Field output* ➔ Hit *Continue…* ➔ Expand *LE*

Check the box *LE33*. If we didn't use Nlgeom then this would be *E33* or strain in the ZZ direction. But when using Nlgeom, this is now a logarithmic strain. Positioning can be changed but we will use the default of *Integration Point*.

Expand upon the Stress section and check the box for S33.

Switch to the *Elements/Nodes* tab and pick the elements of interest. In this case, we select a section of elements close to the center of the compressive element.

**This method does not like too many elements being probed especially when there are 5 steps. Try to keep the element selection count to less than 500. Additionally, it can be helpful to hit the *Active Steps/Frames…* button and only check the steps necessary to create the plot.

Now hit the *Save* button at the bottom of the window. Exit the window.

Tools ➔ *XY Data* ➔ *Manager …* ➔ click the *Create* button ➔ *operate on XY data* ➔ *Continue…*

Now we need to create an equation. Using the built in functions, select *combine(x,x)*. The first x entry will be *average*(all strain entries) and the second x will be *average*(all stress entries). So it looks like this:

combine(average(all strain entries), *average*(all stress entries)*)*

In order to add the entries to the equation, highlight the entries of interest and hit the *Add to Expression* button (Figure 15). Do not forget to add the comma between the strain and stress averages (highlighted in red above).

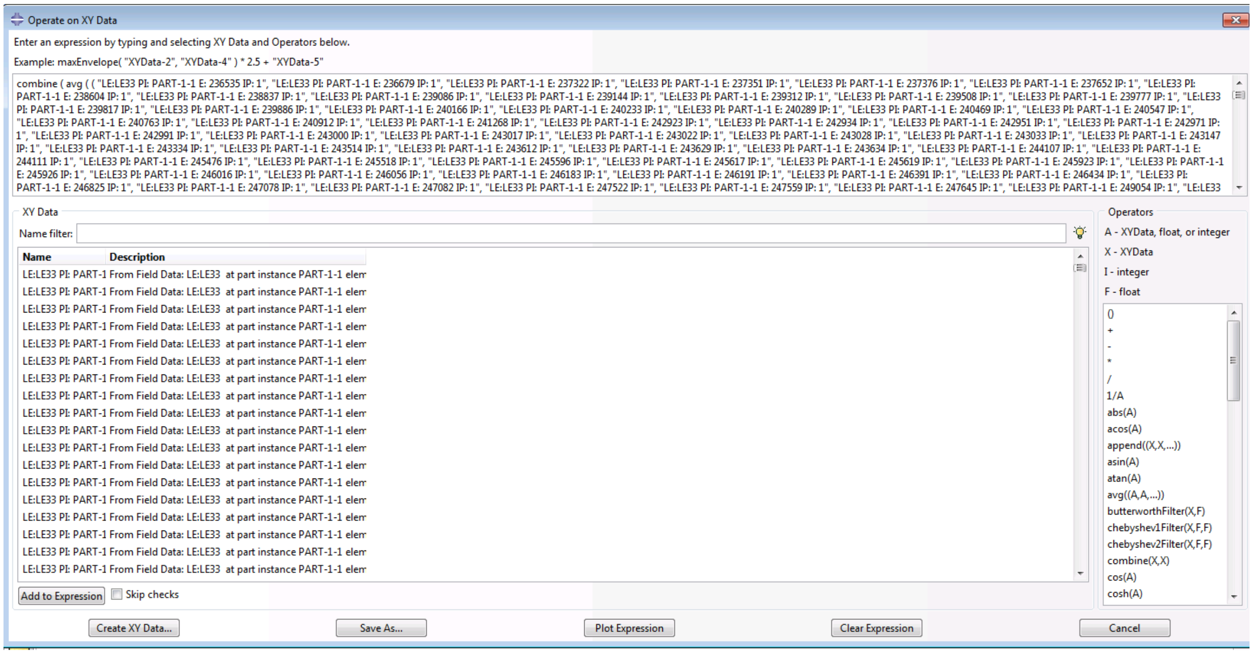

Figure 15: Operate on XY Data to create Stress-Strain Plot

Hit *Plot Expression*. Now using *File* ➔ *Print* a nice TIFF file can be made to capture the newly created plot. Or more useful:

Report ➔ Click *XY plot in current viewport* ➔ click on plot so that it is highlighted ➔ Switch tabs to the *Setup* tab. Select desired directory and settings. Hit *OK*. This gives you the raw averaged data over the steps in an .rpt file (easily imported into Excel).

**In order to exit the plot from the viewport and get back to the 3D model, just hit the plot contours button.

In conclusion, this has created a stress-strain plot that takes an average strain and average stress of a selection of elements throughout each step desired. For this model, this allows a collection of stress-strain responses of the NiTi during the whole model (programming and gait load).

<u>Determine Free Body Loading Diagram</u>

Create a view cut of desired position. In the *View Cut Manager*, select the 4th button on type of model cut. This activates the free body information. To gather just components or just moments, use the free body options. This allows modification to the font and so forth seen in Figure 16.

Options ➔ *Free body…*

**Remember that whatever is displayed in the viewport is playing a role in the *Free Body* calculation. So, if one wanted to know only the load in the nail, use the *Display Group Manager* to only plot the nail jacket.

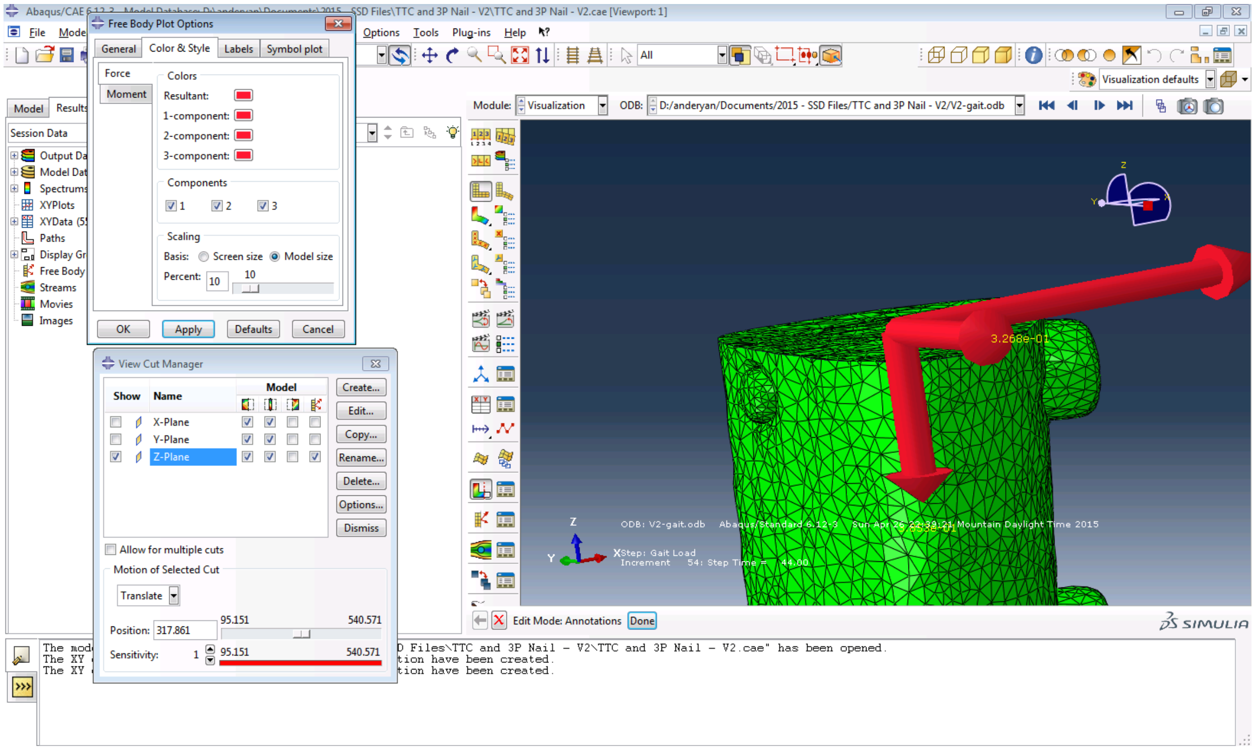

Figure 16: Free Body Cut

<u>Changing Viewport Formatting</u>

Often times it is useful to have a larger legend or change the way it is displayed.

Viewport ➔ *Viewport Annotation Options…*

To change the background in the viewport:

View ➔ *Graphics Options …*

<u>Creating a Movie for Gait Load</u>

Animate ➔ *Time History* ➔ Select this

Options ➔ *Animation …* ➔ *Scale Factor/Harmonic* Tab ➔ Change to *Full Cycle*
➔ Change *Frames* to 44
➔ *Time History* Tab ➔ *Time based*

➔ *Viewports* Tab ➔ *Animation type* : *Time History*

**If looking at the von mises stress (default), modifying the limits can help to highlight change in stress between each frame.

Once the animation looks good and the viewport is to one's liking:

Animate ➔ *Save as…*

The presented methods represent work that was accomplished by a graduate student. By no means is this a definite method to reach the end goal of modeling this device or material. There are numerous ways that this model can be altered or approached to analyze the problem at hand.

GOODLUCK!
