# Supplementary material for: Effect of intramedullary nail stiffness on load-sharing in tibiotalocalcaneal arthrodesis: A patient-specific finite element study
Source: PLoS One. 2023 Nov 16;18(11):e0288049. doi: 10.1371/journal.pone.0288049 (PMC10653524; doi:10.1371/journal.pone.0288049)
Supplement: Attachment — Submitted filename: 2023-0313_PONE-D-23-03456 Review_blind.docx [file pone.0288049.s003.docx]

**PONE-D-23-03456**

**Effect of Intramedullary Nail Stiffness on Load-sharing in Tibiotalocalcaneal Arthrodesis: A Patient-Specific Finite Element Study**

Terrill et al.

**Comments to Authors:**

In this manuscript, the authors built a finite element hindfoot model to determine the influence of different intramedullary nails used for tibiotalocalcaneal arthrodesis on the amount of bone load-sharing with and without different bone resorption depths. Overall, the manuscript is well written, but there are some concerns related to the boundary conditions of the model and its ability to appropriately investigate the devices of interest.

*Introduction*

Lines 48-50, Page 3: A clear and concise explanation of “dynamization of the arthrodesis construct’ should be included prior to or within this sentence to help explain this concept before moving on.

Lines 52-57, Page 3: You state that important factors are maximizing bone-to-bone contact across the fusion site and minimizing micromotion of the joint for proper fusion. You also mention that the device should be rigid to prevent excessive bending and torsional moments at the TTC complex.

How does bone-to-bone contact changes with these different devices?

How does the micromotion change?

Lines 64-66, Page 4: The language should change here to emphasize the comparison of different IM nails here because some of the authors of this manuscript have previously published at least one other study quantifying the load-sharing of arthrodesis devices and bone at the fusion sit, as I understand it.

Lines 78-80, Page 4: For (1), be more specific. The authors are not testing a hypothesis for any material implemented. They are testing a hypothesis for two specific materials. Define ‘high stiffness’. Why not just stick to the second part of the hypothesis? The introduction leads into the second part of the hypothesis, but the first part seems out of place or at least not supported in the Introduction.

*Method*

Lines 99-100, Page 5: The study is interesting, but the single, healthy subject used to develop the finite element model without experimental validation limits the impact.

Lines 112-113, Page 5: Why not demonstrate the influence of different IM nails with varying material properties, since only one subject was used to develop a single finite element model? In the previous study by the authors, they vary the effect of bone modulus and demonstrate changes. How does the comparison in this manuscript of the different IM nails changes in relation to the bone modulus, and does the positive effect of the dynamization decrease with different bone quality?

Lines 129-130, Page 6: Why not demonstrate the influence from different IM nails with different alignment? How sensitive are the results to changes in position and alignment of the device?

Lines 132-135, Pages 6-7: Where does the coefficient of friction value come from?

Lines 132-135, Pages 6-7: Is the bone-nail interface perfect? Is that realistic? What implications are there for modeling the IM nails this way? This model representation negates the ability of the device to settle with further loading and potentially change the results. This should probably be mentioned in the Discussion.

Lines 135-136, Page 7: Do you mean frictionless here? The language used in this sentence does not make this clear.

Lines 142-143, Page 7: I would refer to this as ‘simulated resorption behavior’ because the representation of resorption is simplified.

Lines 164-166, Page 8: I am confused by the loading applied. How can you apply this load to just the distal end of the calcaneus where the IM nail was implanted and call it ‘gait loading’? What about during toe-off when the ground reaction force is being applied to the forefoot, which wasn’t modeled? Also, why weren’t the off axis loads applied to assess the complexity of loads during stance? The loading does not seem physiologic if all the load is always applied to the distal calcaneus near the IM nail implanted.

*Results*

Lines 197-198, Page 9: Change ‘no gait loading’ and ‘peak gait load’ to ‘unloaded’ and ‘peak compressive load’. This manuscript may be basing the compressive force off of the ground reaction forces during gait, but they are only being applied as a range of axial compressive loads. They do not replicate the loads under walking conditions, as these would be more complex.

Lines 201-210, Page 10: Why focus on reporting absolute values? Since you only have one subject without any experimental validation, the analysis should consist of relative comparisons between the different nails. There is no ability to determine if these values are correct, or that they represent the values that would be demonstrated in others.

Lines 205-206, Page 10: Why did you compare 400 to 500N? If you can’t achieve 500N with the NiTi nail, then why not just compare each to 400N? I understand that the differences found between the two are vast, even with this discrepancy, but the inconsistency in methodology doesn’t make sense.

Lines 217-224, Pages 10-11: The simulations seem to be quasistatic and not dynamic, so I’m not sure why two cycles of gait loading were performed. It seems like the axial load was just varied. Were dynamic simulations performed and acceleration of loading during walking included?

Lines 232-234, Page 11: It is not clear where at ‘toe-off’ you are referring to make this calculation.

Lines 253-264, Page 12: I like the sensitivity analysis of simulated bone resorption in this study. Why not just focus on this aspect? Since this is one model of a single foot without experimental validation, the sensitivity of different amounts of bone resorption is more interesting and impactful than the earlier analysis.

*Discussion*

Lines 310-313, Page 14: Can you really say that this is dynamic compressive loading? It is not clear from the methods that you can. It appears you applied different values of compressive load near the site of the nail.

Lines 313-315, Page 14: I would use ‘suggest’ instead of ‘exhibit’ here. The language is a little strong for an analysis with one model.

Lines 369-372, Page 17: Why not include more specimens then to present results in light of different subject variability in bone material properties, geometry, alignment, etc.?

Lines 372-374, Page 17: This is true, but the finite element model would be more impactful with some level of experimental validation and then use this to even extrapolate predictions of stress.
