# Supplementary material for: Effect of intramedullary nail stiffness on load-sharing in tibiotalocalcaneal arthrodesis: A patient-specific finite element study
Source: PLoS One. 2023 Nov 16;18(11):e0288049. doi: 10.1371/journal.pone.0288049 (PMC10653524; doi:10.1371/journal.pone.0288049)
Supplement: Attachment — Submitted filename: 2023-0513_PONE-D-23-03456.R1 Review_blind.docx [file pone.0288049.s005.docx]

**PONE-D-23-03456.R1**

**Effect of Intramedullary Nail Stiffness on Load-sharing in Tibiotalocalcaneal Arthrodesis: A Patient-Specific Finite Element Study**

Terrill et al.

**Comments to Authors:**

*Response to Author Responses to Reviewers*

Although the authors state that their methodology allows them to isolate the effects of the IM nails with their one subject, which is true, it still does not eliminate the need to perform the same analysis in other subject models to support their conclusions. A repeated-measures analysis with multiple subject models does not prevent an investigation to isolate the effects of IM nails. In fact, I think it is important to analysis different aspects that these nails will encounter as a part of a sensitivity analysis in order to say anything valuable about the results. If multiple models generated with different geometry and bone material properties could exhibit the behavior that the authors have stated, then this would improve the impact of their analysis. The problem with this study is that the authors have only found that one subject had the effects they describe, where we don't know whether this is the case with other subjects or with other conditions that are possible like malalignment or reduced bone density. This should at least be addressed further in the limitation section as this is more work, but these details may influence their conclusions.

*Introduction*

Lines 81-86, Page 4: Include background information and rationale within the earlier paragraphs of the Introduction for evaluating nails with bone resorption modeled. There was no mention of bone resorption until the objective and hypothesis. Please elaborate to prepare the reader for the objective and hypothesis.

*Methods*

Line 182, Page 9: Change “peak gait load” to “peak compressive load.” You are applying a compressive force from a value extracted from the ground reaction force and applied to the opening of the implanted nail. You are not representing gait loading. Change this throughout the manuscript.

Line 182-184, Page 9: Do you mean “vertical ground reaction force”? Is that where you are getting this value? Same for Lines 187-188.

*Results*

Lines 242-244, Page 12: Two cycles of gait loading? This is confusing because these are quasistatic simulations. Do you mean that you are running multiple simulations or steps while varying the compressive load applied? If so, clarify as such within the results (even though this is more of a method detail).
