# Supplementary material for: Effect of intramedullary nail stiffness on load-sharing in tibiotalocalcaneal arthrodesis: A patient-specific finite element study
Source: PLoS One. 2023 Nov 16;18(11):e0288049. doi: 10.1371/journal.pone.0288049 (PMC10653524; doi:10.1371/journal.pone.0288049)
Supplement: Attachment — Submitted filename: Response to Reviewers.docx [file pone.0288049.s006.docx]

**Response to Reviewers**

The authors would like to thank the reviewer for their time and effort in thoughtfully evaluating our revised manuscript. Below we have included each question/comment provided by the reviewer along with our responses. The Revised Manuscript with Track Changes document highlights the changes made in response to the critiques, and the changes are described below. Please note that the line numbers listed in our italicized responses below correspond to the line numbers in the marked up copy. We again thank the reviewer for identifying the need for these changes that have resulted in a stronger paper, which we hope is now suitable for publication in PLOS ONE.

**Comments and Responses:**

Response to Author Responses to Reviewers

Although the authors state that their methodology allows them to isolate the effects of the IM nails with their one subject, which is true, it still does not eliminate the need to perform the same analysis in other subject models to support their conclusions. A repeated-measures analysis with multiple subject models does not prevent an investigation to isolate the effects of IM nails. In fact, I think it is important to analysis different aspects that these nails will encounter as a part of a sensitivity analysis in order to say anything valuable about the results. If multiple models generated with different geometry and bone material properties could exhibit the behavior that the authors have stated, then this would improve the impact of their analysis. The problem with this study is that the authors have only found that one subject had the effects they describe, where we don't know whether this is the case with other subjects or with other conditions that are possible like malalignment or reduced bone density. This should at least be addressed further in the limitation section as this is more work, but these details may influence their conclusions.

*We agree with the reviewer and do appreciate the value that would be added by analyzing the nail behavior for a range of different subjects’ ankles. We do not have reason to believe that the relative nail behaviors would not be reproduced in other subjects, but we agree that additional analyses would need to be performed in order to quantitatively confirm this. However, given the added complexity in what is already a quite complex set of models (note that we added an 18-page detailed description of the methodology to the supporting information), at this time a comparison across different subjects is out of the scope of the current study. We have addressed this limitation further in the discussion (lines 415-421) and have added a qualifying statement to the conclusions (line 426).*

Introduction

Lines 81-86, Page 4: Include background information and rationale within the earlier paragraphs of the Introduction for evaluating nails with bone resorption modeled. There was no mention of bone resorption until the objective and hypothesis. Please elaborate to prepare the reader for the objective and hypothesis.

*We have added verbiage and supporting references to raise the issue of localized resorption in the introduction (lines 60-65).*

Methods

1. Line 182, Page 9: Change “peak gait load” to “peak compressive load.” You are applying a compressive force from a value extracted from the ground reaction force and applied to the opening of the implanted nail. You are not representing gait loading. Change this throughout the manuscript.

*We have made this change and removed the word “gait” where appropriate (lines 178, 189, 223,	228, 229, 238, 240, 248, 253, 260, 262, and the captions for figures 5, 6, and 7).*

2. Line 182-184, Page 9: Do you mean “vertical ground reaction force”? Is that where you are getting this value? Same for Lines 187-188.

*We have changed the wording to “maximum vertical ground reaction forces” in lines 179-180	and “applied load” in line 189.*

Results

Lines 242-244, Page 12: Two cycles of gait loading? This is confusing because these are quasistatic simulations. Do you mean that you are running multiple simulations or steps while varying the compressive load applied? If so, clarify as such within the results (even though this is more of a method detail).

*For added clarity, we have added an explanation for why we used two cycles (due to the different behavior of NiTi in shortening and lengthening) and have added an explanation of the multiple quasistatic time points (lines 238-244). In order to clarify that the FEA is not in fact dynamic, we also removed the word* dynamic *from lines 256, 346, 352, 356, 366, and 385, and from the caption for Fig. 6.*
